# High-throughput full-length single-cell mRNA-seq of rare cells

**DOI:** 10.1371/journal.pone.0188510

**Published:** 2017-11-29

**Authors:** Chin Chun Ooi, Gary L. Mantalas, Winston Koh, Norma F. Neff, Teruaki Fuchigami, Dawson J. Wong, Robert J. Wilson, Seung-min Park, Sanjiv S. Gambhir, Stephen R. Quake, Shan X. Wang

**Affiliations:** 1 Department of Chemical Engineering, Stanford University, Stanford, California, United States of America; 2 Department of Bioengineering, Stanford University, Stanford, California, United States of America; 3 Department of Life Science and Applied Chemistry, Nagoya Institute of Technology, Nagoya, Japan; 4 Department of Electrical Engineering, Stanford University, Stanford, California, United States of America; 5 Department of Materials Science and Engineering, Stanford University, Stanford, California, United States of America; 6 Department of Radiology, Stanford University School of Medicine, Stanford, California, United States of America; 7 Molecular Imaging Program at Stanford, Stanford University School of Medicine, Stanford, California, United States of America; 8 Canary Center at Stanford for Cancer Early Detection, Stanford University School of Medicine, Palo Alto, California, United States of America; 9 Department of Applied Physics, Stanford University, Stanford, California, United States of America; 10 Chan Zuckerberg Biohub, San Francisco, California, United States of America; The Ohio State University, UNITED STATES

## Abstract

Single-cell characterization techniques, such as mRNA-seq, have been applied to a diverse range of applications in cancer biology, yielding great insight into mechanisms leading to therapy resistance and tumor clonality. While single-cell techniques can yield a wealth of information, a common bottleneck is the lack of throughput, with many current processing methods being limited to the analysis of small volumes of single cell suspensions with cell densities on the order of 10^7^ per mL. In this work, we present a high-throughput full-length mRNA-seq protocol incorporating a magnetic sifter and magnetic nanoparticle-antibody conjugates for rare cell enrichment, and Smart-seq2 chemistry for sequencing. We evaluate the efficiency and quality of this protocol with a simulated circulating tumor cell system, whereby non-small-cell lung cancer cell lines (NCI-H1650 and NCI-H1975) are spiked into whole blood, before being enriched for single-cell mRNA-seq by EpCAM-functionalized magnetic nanoparticles and the magnetic sifter. We obtain high efficiency (> 90%) capture and release of these simulated rare cells via the magnetic sifter, with reproducible transcriptome data. In addition, while mRNA-seq data is typically only used for gene expression analysis of transcriptomic data, we demonstrate the use of full-length mRNA-seq chemistries like Smart-seq2 to facilitate variant analysis of expressed genes. This enables the use of mRNA-seq data for differentiating cells in a heterogeneous population by both their phenotypic and variant profile. In a simulated heterogeneous mixture of circulating tumor cells in whole blood, we utilize this high-throughput protocol to differentiate these heterogeneous cells by both their phenotype (lung cancer versus white blood cells), and mutational profile (H1650 versus H1975 cells), in a single sequencing run. This high-throughput method can help facilitate single-cell analysis of rare cell populations, such as circulating tumor or endothelial cells, with demonstrably high-quality transcriptomic data.

## Introduction

In recent years, much work on technologies and chemistries for enrichment of biological cell subpopulations, and subsequent single-cell level analysis, has emerged [1–4]. Among other achievements, this has led to the discovery of rare subpopulations such as tumor-initiating cells in solid and hematopoietic tumors [5, 6]. Work by Yu et al. and Miyamoto et al. are striking examples of how researchers utilized single-cell measurements to characterize heterogeneity in response to cancer treatment, and illustrate how single-cell RNA-seq can deliver insights into pathways in therapy-related resistance in cancer [4, 7, 8].

While the wealth of information is a big driver for single-cell characterization, the subpopulation of interest in many situations is an extremely scarce component of the entire bulk population, rendering rapid isolation and preparation of these rare cells for single-cell analysis as much of a challenge as the actual single-cell sequencing. The human circulatory system, in particular, consists of many interesting cell subpopulations, such as hematopoietic stem cells, relevant in recovery from marrow ablative therapy [9], and activated immune cells in cancer immunotherapy [10]. Similarly, stem cell populations in solid tumors can be as scarce as 0.01% [11], while circulating tumor cells (CTC) are present in the whole blood of diseased patients at cell concentrations of 1–10 parts per billion [12–15].

In many single-cell studies, fluorescence-activated cell sorting (FACS) remains the laboratory technique of choice for enrichment of the rare subpopulation, as it can achieve single-cell separation on multiple cell markers and is a relatively mature technology [16, 17]. Additionally, immuno-fluorescence reagents for FACS are widely available commercially. Nonetheless, the technology faces a fundamental limitation due to its serial processing. Ultimately, every cell has to be interrogated sequentially as it passes the optical apparatus, and every cell must be deflected separately into the appropriate receptacle (e.g. a 96-well microplate). An event rate of 10^4^ /s is cited as the practical upper limit for FACS due to the high pressures required for faster flow-rates being detrimental to cell viability [18]. Barring massive parallelism, this results in sort times on the order of hours for a population of 10^7^ cells, and this linear scaling makes sorting samples such as whole blood, with > 10^9^ cells / mL, impractical without prior processing.

The need for rapid, high through-put cell isolation techniques is further emphasized by the relatively fast decay rates of human mRNA, with their median half-life of 10 hours [19]. Essentially, extended processing times can result in mRNA profiles being measured that are different from the actual time of sampling, further confounding the testing of biological hypotheses [20].

Hence, many researchers have innovated various devices for rapid cell enrichment, both as a pre-processing step for integration with single-cell platforms such as Fluidigm’s C1 and Biomark machines, or for direct single-cell characterization on-chip [21–25]. Nonetheless, a majority of these devices leverage on microfluidic technology, which can present significant practical difficulties when large sample volumes are required. On the contrary, the magnetic sifter, which utilizes standard MEMS processing for easy fabrication, yet is 3-dimensional in operation, allows for high-throughput via fast volumetric flow-rates [26], while leveraging on the high specificity of immuno-magnetic cell separation, as demonstrated in other immuno-magnetic flow-through cell separation systems [27–29]. Having previously presented its application to the enrichment and enumeration of CTC on-chip [26], we further demonstrate the ease of cell recovery post-enrichment by the sifter, and apply it, in combination with FACS, to obtain high-quality single-cell expression data by the Smart-seq2 protocol.

We evaluate our method with 2 non-small-cell lung cancer (NSCLC) cell lines (NCI-H1650 and NCI-H1975 from ATCC, Manassas, VA), and illustrate the ease with which this protocol can be adapted towards identifying distinct cell populations in a simulated heterogeneous mixture. We then present a heuristic for analyzing single-cell mRNA-seq data for mutations and gene expression differences based on our cell line data, which can be useful to researchers interested in simultaneous analysis of genotype-phenotype data. Lastly, while we applied this method towards the isolation and analysis of simulated circulating tumor cells in blood, the flexibility of this approach allows easy adaptation towards other systems where rapid isolation of rare cells from a highly heterogeneous matrix is required, such as in the isolation of a specific subcomponent of the human immune system.

## Results

### Protocol efficiencies

**Fig 1:**
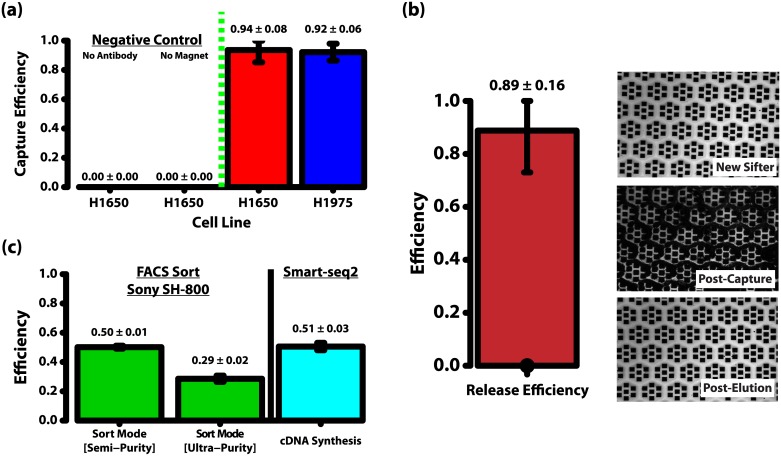
Efficiencies of different steps in this method. (a) The sifter shows high capture efficiencies (> 90%) for 2 NSCLC cell lines tested (H1650 and H1975). Additionally, 2 sets of negative controls were also done with H1650 cells, with no non-specific capture observed. These negative controls are run as per the regular experiments, but with non-antibody functionalized magnetic nanoparticles (negative control for non-specific nanoparticle capture), and without the application of a magnet (negative control for non-magnetic capture). (b) The sifter also exhibits good release properties of captured cells and magnetic nanoparticles (89%). Optical images illustrate the effectiveness of elution from the magnetic sifter. The sifter surface post-elution appears as pristine as the surface of a brand new sifter. (c) FACS sort efficiencies vary with sort purity settings. 2 sort settings on the Sony SH-800 cell sorter are tested. Efficiencies of 50% and 29% are observed for the semi-purity and ultra-purity modes respectively. A reduced purity setting is required for higher yields. By following the Smart-seq2 protocol exactly, we observed successful cDNA synthesis in 51% of the wells.

Spiked NCI-H1650 cells were added to healthy donor blood from the Stanford Blood Center, isolated with anti-Epithelial Cell Adhesion Molecule (EpCAM) functionalized MNPs (NVIGEN, Inc, Sunnyvale, CA), sorted by FACS as single cells into 96-well plates (Sony LE-SH800 cell sorter, Sony Biotechnology, San Jose, CA), and then prepared for sequencing as per the Smart-seq2 protocol [30]. At every step, the cells were counted to evaluate the efficiencies associated with every process. Measured efficiencies are shown in Fig 1. Capture efficiency on the magnetic sifter for NCI-H1975 cells spiked into blood is also presented to illustrate the consistency in sifter capture performance.

Capture efficiencies were evaluated with 2 NSCLC cell lines, H1650 and H1975 cells, and both showed good capture performance on the magnetic sifter (94% and 92% respectively), as shown in Fig 1(a). Crucially, release efficiencies of the NSCLC cells from the magnetic sifter were consistently high, with an average of 89% as per Fig 1(b). This is especially pertinent in rare cell isolation, where cell losses need to be minimal.

From Fig 1(c), it is clear that cell losses associated with this protocol are primarily due to the FACS sort, while the standard Smart-seq2 chemistry is only 51% efficient on a 96-well microplate in this work. These processes were done with standard instrument settings (for FACS), and published protocols (for Smart-seq2), and were not further optimized in this work, indicating the potential for higher overall efficiencies. However, since FACS involves a trade-off between sample purity (probability of each droplet/well containing only single-cells) and sample yield (percentage of droplets/cells discarded), even if further improvements in yield are possible, concerns about purity may not make it desirable.

In this instance, if the semi-purity mode is used for the sort, the entire protocol would result in a final yield of 20%. This is similar to the 20% yield reported by Swennenhuis et al. when they combined the FDA-cleared CellSearch system with whole genome amplification for the analysis of circulating tumor cells [31].

### Gene expression analysis

Using this protocol for isolation and sequencing of rare cells in blood, we sequenced H1650 single cells isolated from healthy donor blood in 3 separate runs of a simulated CTC experiment, and compared the results to sequencing results from bulk H1650 cells that had been freshly harvested from a tissue culture dish, and bulk white blood cells (WBCs) from healthy donor blood. This was done to verify that the transcriptomic data obtained from H1650 cells post-magnetic sifter separation continues to resemble the starting bulk populations.

**Fig 2:**
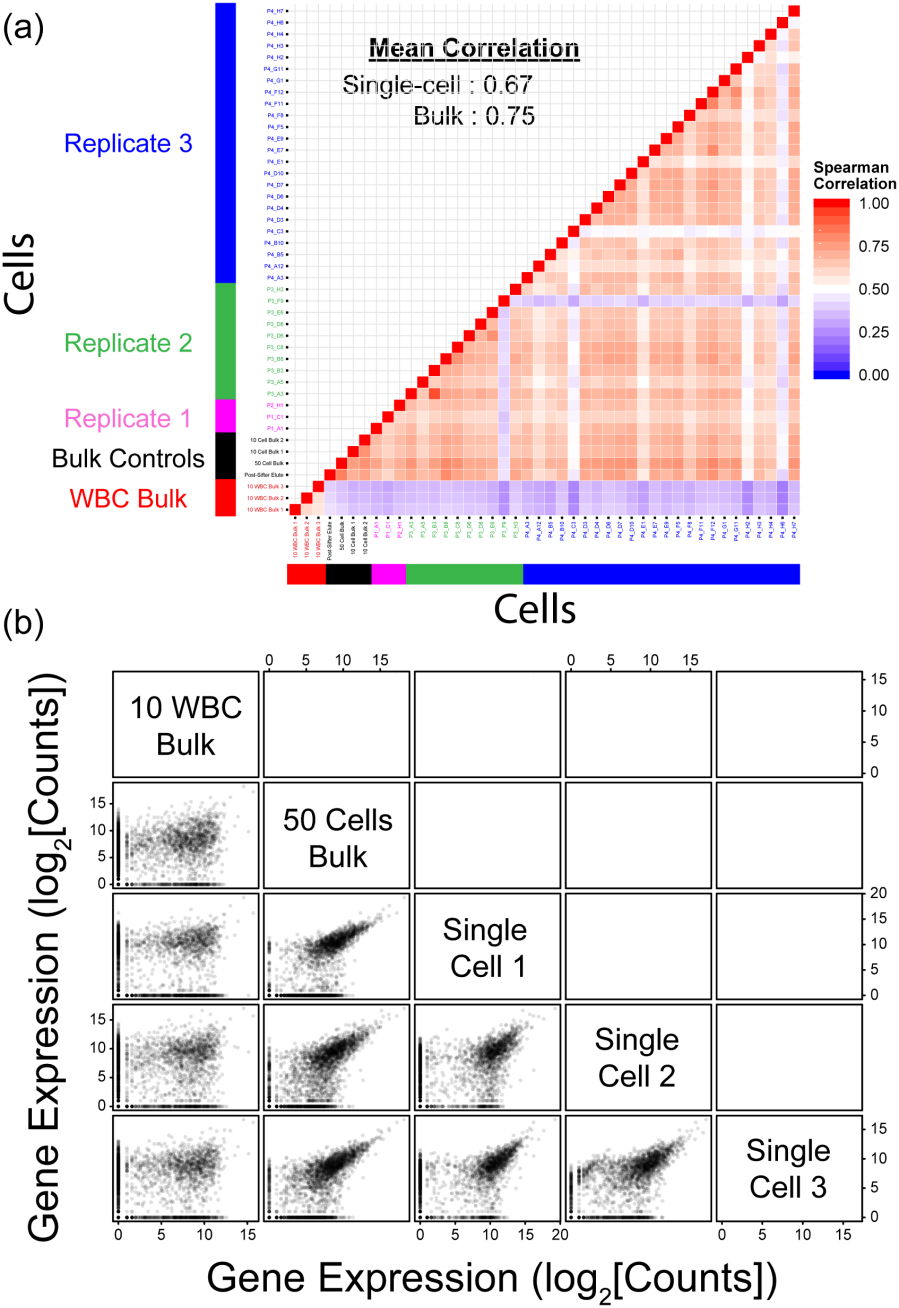
(a) Gene expression correlations between single cells from replicate experiments. The Spearman correlations observed are similar across replicates, and are just slightly lower than bulk controls (0.67 vs 0.75). In contrast, when these single-cell H1650 transcriptomes are compared to those of white blood cells, very low correlation is observed. Color of axis labels illustrate specific sample types, with red representing bulk white blood cell samples, black representing bulk H1650 cell samples, and purple, green and blue representing single H1650 cells from 3 separate replicate experiments. (b) Sample scatter plots illustrating correlation in gene expression between white blood cells (WBCs) and H1650 cells. The single-cell data (3 randomly chosen examples shown) match the H1650 bulk sample (50 cells bulk), while having little correlation with the bulk WBC sample (10 WBC bulk).

A subset of isolated cells was selected from each run for library preparation to minimize cost. Libraries were also prepared from wells containing more than 1 H1650 cell (termed bulk H1650 samples). The single-cell gene expression data obtained from 3 separate runs was then compared to bulk H1650 cells and WBCs separately FACS-sorted from donor blood. Pair-wise Spearman’s correlation was computed for the gene expression across all single cells and bulk cells, as a measure of their similarity, and the inter-cell correlations in Fig 2(a) show that the transcriptomes obtained between single cells after sifter processing remain similar across runs. When averaged across all pair-wise combinations, inter-cell correlations for the single H1650 cells are 0.67 ± 0.1, while the correlations for the bulk H1650 samples are 0.75 ± 0.04. There appear to be outliers in some of the single cells analyzed, with transcription patterns that do not match either white blood cells or the other H1650 cells. However, in the absence of further analysis to understand the biological reason for these outliers, they have not been excluded from the calculation of inter-cell correlation. The current value of 0.67 is hence anticipated to be higher if these outliers are removed. Nonetheless, the close match between the single-cell and bulk measurements and similarity of the former to literature values of 57% and 65% for single-cell variability further validates this protocol [32–34]. Additionally, the H1650 samples exhibit poor correlation with the WBC samples. The results illustrate good reproducibility of data obtained by isolation with the magnetic sifter, and show that the protocol can provide high quality and consistent transcriptomic data. Sample scatter plots are also shown in Fig 2(b), illustrating the good correlation between the individual H1650 single cells and the bulk H1650 sample, and the lack of any correlation when compared to the WBC samples.

**Fig 3:**
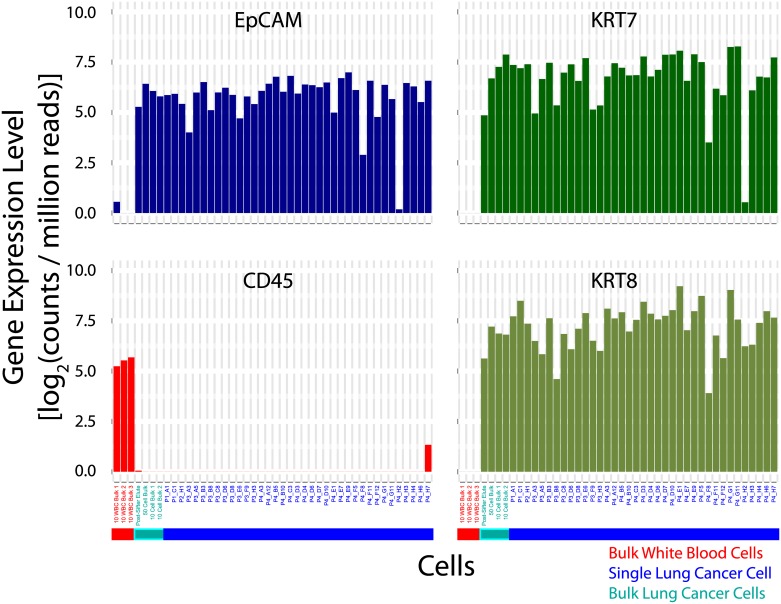
Epithelial and WBC gene expression levels. Clear differences in *CD45* (WBC marker) and *EpCAM/KRT7/KRT8* (epithelial) genes are observed between the white blood cells and the H1650 cells.

In addition, since we are simulating a CTC system, we analyzed the genes commonly used to discriminate putative CTCs from WBCs in immunohistochemistry [35, 36]. Previous work has shown that cytokeratins 7 and 8 (*KRT7* and *KRT8*) can be targeted in lung adenocarcinomas, while white blood cells should have no cytokeratin expression [26, 37, 38]. In addition, *CD45* is a common white blood cell marker that should not be present on epithelial cells. Hence, we looked at the expression levels of this panel of 4 genes (*EpCAM*, *KRT7*, *KRT8*, *CD45*), to verify that we can successfully identify the cells as being of epithelial origin, as plotted in Fig 3.

It is clear that the majority of the isolated cells are transcriptionally epithelial in nature, with high *EpCAM*, *KRT7* and *KRT8* expression, and no *CD45* expression, although 2 of the 37 cells evaluated do exhibit atypical profiles. This matches the results from bulk H1650 cells, and is the opposite of sequenced WBCs, which only exhibit *CD45* expression.

### Mutational analysis from mRNA-seq data

Typically, in sequencing experiments, the experimenter has to make an upfront decision to focus on either genomic or transcriptomic data. Single-cell genomes can provide genetic heterogeneity and cell-lineage information, while single-cell transcriptomes can help define the cells’ current phenotypes. However, in many instances, both sets of information are of interest to the experimenter, and interactions between the genotype and phenotype can be illuminating. While this can be circumvented in bulk experiments by up-stream division of the sample into two components, this is not possible in rare cell populations, where the amount of starting material is scarce. Currently, many researchers are working on methods to accomplish simultaneous genomic and transcriptomic sequencing, however, these methods can be relatively complicated [39]. A simpler work-around in literature is to utilize mRNA-seq data for information on genetic heterogeneity, although mRNA-seq data is still primarily used for expression-level analysis [40, 41]. Hence, we explore the possibility of using mRNA-seq data from Smart-seq2 to gain insights into variants in the cells isolated, as it was hoped that this method would provide both mutational and expression-level data simultaneously in a more economical and informative experimental setup.

Additionally, Picelli et al. previously demonstrated the ability of the Smart-seq2 protocol to generate full-length mRNA-seq data [42]. This is particularly useful for obtaining genetic level information from transcriptomic data as we hypothesized that any nucleotide position in the exons of genes being expressed will have equal probability of being sequenced, with a scaling factor reliant on the gene's expression level. Essentially, we should be able to observe mutations across entire gene isoforms, as opposed to only mutations at the 3’-end of the mRNAs with the use of a full-length mRNA-seq protocol.

With our simulated CTC system, we can evaluate our ability to obtain mutational information from mRNA-seq data. We first extracted a list of common mutations in the H1650 cell line from the Catalogue of Somatic Mutations in Cancer (COSMIC) [43, 44]. We then narrowed this list to single nucleotide polymorphisms (SNPs) in exonic regions, and looked for this list of SNPs in the transcriptomic data.

**Fig 4:**
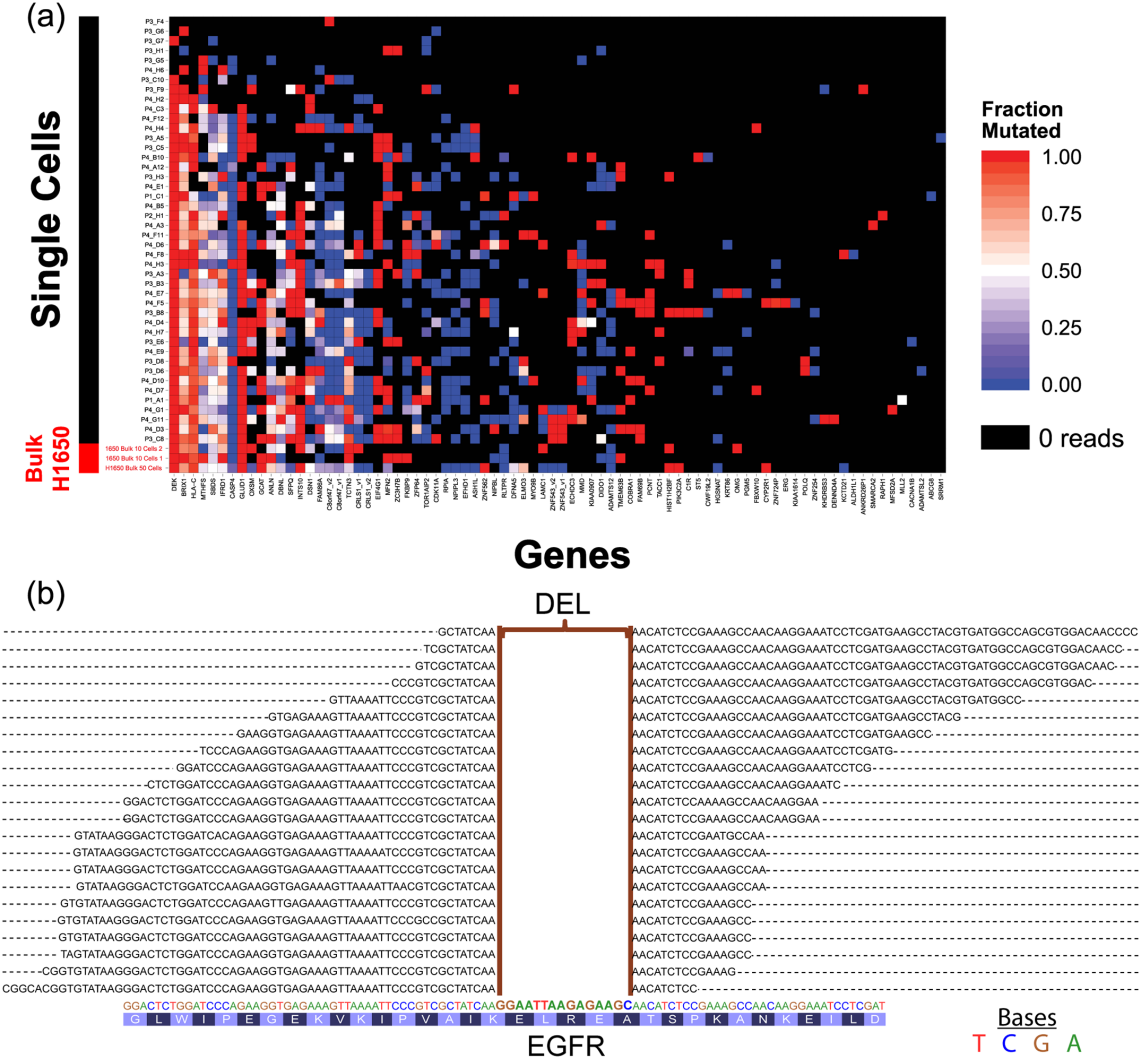
(a) Observed COSMIC SNPs in H1650 transcriptional data. SNPs can be identified in the H1650 cells after being processed through the magnetic sifter. Only 81 of 151 known SNPs had coverage. Not all known SNPs showed up in the transcriptional data, while some genes with coverage showed almost no mutated allele. This could be due to effects such as reduced expression of the mutated allele relative to the wild-type allele. It should be noted that the results are consistent with the bulk H1650 samples, suggesting the SNPs that are not observed are truly not present in the H1650 transcriptome. A full list of the genes is included in S1 Table. (b) *EGFR* exon 19 deletion in H1650 single-cell Smart-seq2 data. The figure shows a collection of unique Smart-seq2 reads spanning the *EGFR* exon 19 region from a single H1650 cell, with a clear deletion in the exon as predicted from the COSMIC database.

Of 151 SNPs from COSMIC, we only observed 81 in the cells' transcriptome, as shown in Fig 4(a). This is not completely unexpected, and highlights the inherent difficulty of attempting to identify mutational level information from transcriptional data. If the gene is not highly expressed, identifying *de novo* mutations with high statistical confidence can be difficult. Also, mutations which cause suppression or inactivation of the mutated allele gene might result in only the wild-type allele being observed. Nonetheless, any mutation detected is still effectively providing additional information over and above what would be typically determined from mRNA-seq data.

H1650 cells are commonly studied in literature for their *EGFR* exon 19 deletion, an especially important driver mutation in NSCLC of interest to clinicians as it can be specifically targeted with therapies such as erlotinib [45–47]. Hence, we also attempted to detect this particular deletion in the H1650 transcriptomic data. By analyzing the individual reads from each cell, we could clearly observe the deletion in the base pairs corresponding to the exon 19 deletion. A collection of the different reads obtained from sequencing that span the *EGFR* exon 19 location are displayed in Fig 4(b), illustrating the actual loss in base pairs in the read sequences. We have thus successfully identified this particular *EGFR* deletion, illustrating that transcriptomic data can be used for detection of both point mutations, as per Fig 4(a), and longer exon insertions or deletions, as per Fig 4(b).

**Fig 5:**
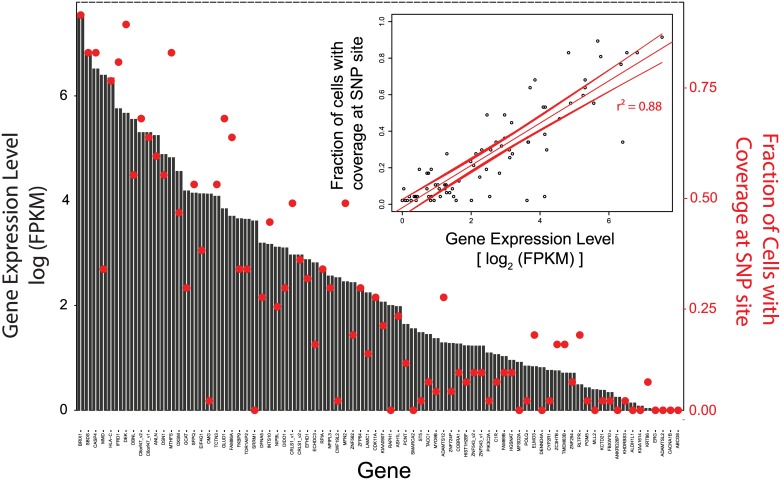
Relationship between SNP coverage and gene FPKM levels. Gene expression levels (log_2_[FPKM]) are compared with the fraction of cells with coverage at every SNP site, and the inset shows the regression curve. A linear relationship is obtained, with an r^2^ of 0.88. A full list of the genes is provided in S2 Table, going from left to right.

In typical mRNA-seq data, gene expression levels are often quantified by the fragment per kilobase of exon per million reads (FPKM). This normalizes the amount of reads for a particular gene by the length of the gene, and the depth of sequencing. Incidentally, this is also a good measure for normalizing the probability of observing a particular base location in the exon of any gene when doing full-length mRNA-seq. Hence, we plotted a linear regression relating the probability of having coverage at a SNP site to the gene expression level, based on the H1650 transcriptomic data, and observed a good linear correlation. Based on the data presented in Fig 5, we further conclude that we have a greater than 50% chance of detecting any SNP within the exon of a gene when the gene has an expression level greater than 16 FPKM. The linearity of this relationship (r^2^ = 0.88) further proves how Smart-seq2 is indeed providing reads that have an equal probability of spanning entire gene isoforms, with no 3’-end bias. This heuristic can also serve as a guideline for defining what constitutes a high enough level of gene expression for observation of SNPs in the transcriptome.

### Observing single-cell heterogeneity in a mixed population

**Fig 6:**
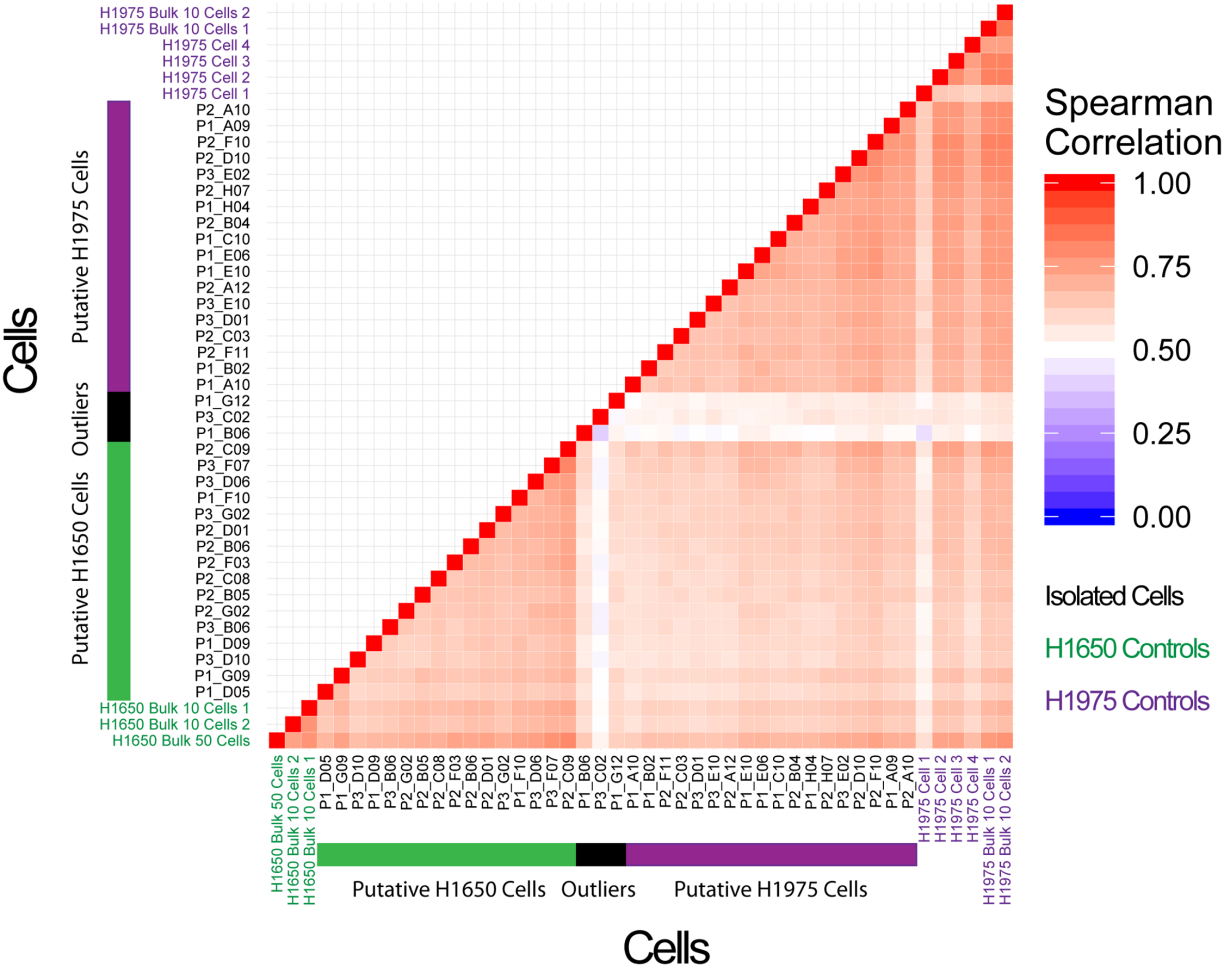
Differentiating simulated CTC subpopulations by gene expression analysis. H1975 and H1650 cells are spiked into blood, isolated by magnetic separation, and analyzed. Their gene expression levels are very similar, and are consistent with prior results on the individual pure populations for both cell lines. Two distinct subpopulations are identified by hierarchical clustering among the isolated cells with p < 0.05, with one being a putative H1650 subpopulation, and the other being a putative H1975 subpopulation.

To further demonstrate the use of mRNA-seq coupled with the magnetic sifter for unraveling single-cell heterogeneity in CTCs, we simulated a mixed CTC population by spiking a 1:1 mix of H1650 and H1975 NSCLC cells into blood. As the 2 cell lines are both NSCLC cells, gene expression levels for all cells isolated and sequenced are very similar. Nonetheless, hierarchical clustering based on their gene expression levels is able to distinguish two distinct clusters of cells (p < 0.05), in addition to three outliers. The pair-wise Spearman’s correlation coefficient between the isolated single cells is shown in Fig 6, and illustrates two distinct putative H1650 and H1975 clusters. The average inter-cell correlation within the putative H1650 clusters and the putative H1975 clusters are 0.62 ± 0.1 and 0.66 ± 0.1 respectively, while the average inter-cell correlation between cells within the putative H1650 and the H1975 clusters is 0.56 ± 0.05, thus further supporting the identification of these two clusters.

**Fig 7:**
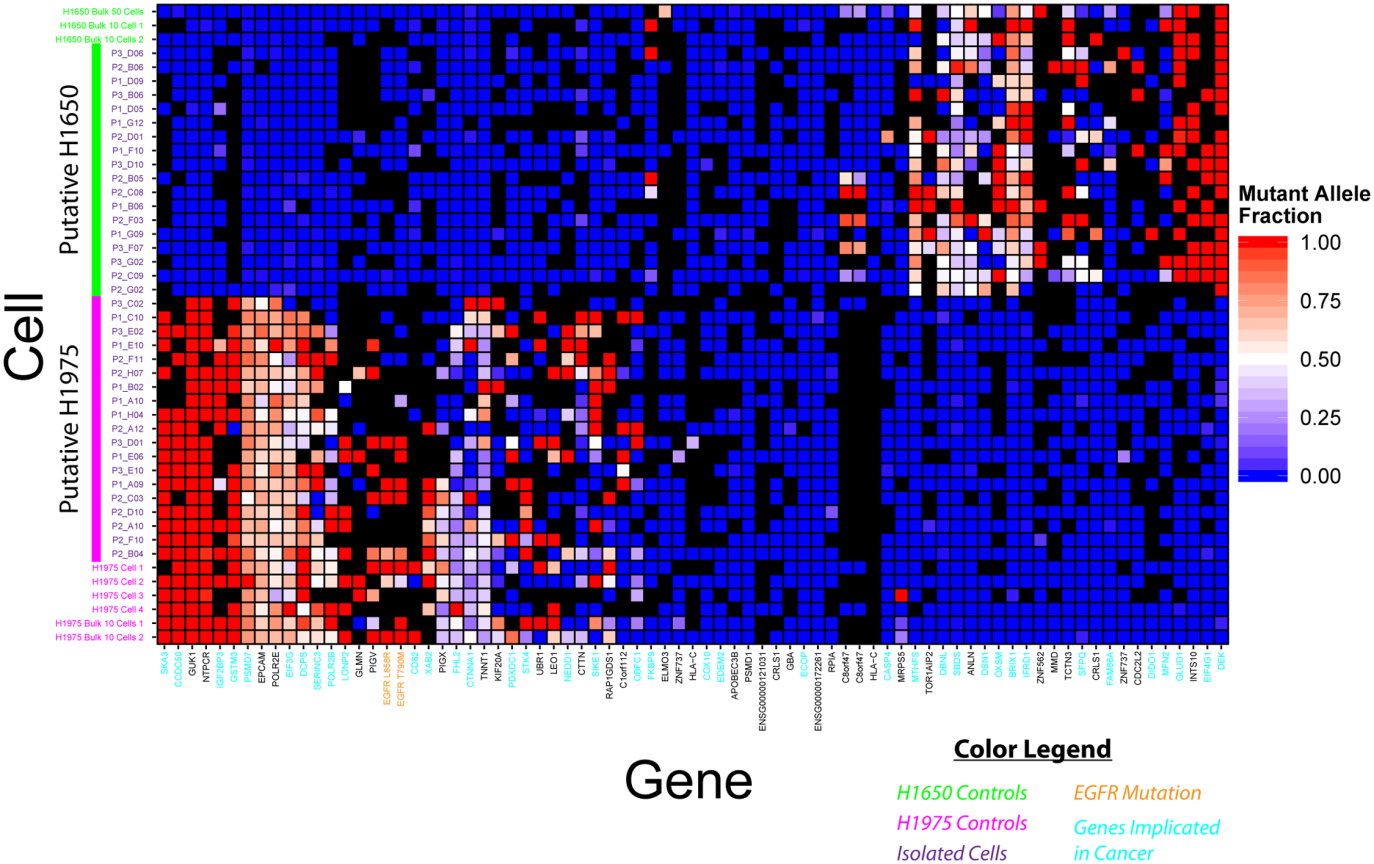
Differentiating simulated CTC subpopulations by mutational analysis. H1975 and H1650 cells are spiked into blood, isolated by magnetic separation, and sequenced. By looking at the variants present in the cells, we are able to observe the same subpopulation mix as were originally spiked into the blood sample. A full list of the genes is provided in S3 Table, going from left to right.

Excitingly, unsupervised clustering of mutational analyses of these same cells picked up the two separate sets of cells that were spiked into the original sample, as shown in Fig 7. The analyzed cell population yielded two clusters that were an almost exact 1:1 mix of putative H1650 and H1975 cells, corresponding to the original ratio of cells that were spiked into the simulated CTC samples. This corroborates the similarity in capture efficiencies for these two cell lines (> 90% in Fig 1), and further illustrates the consistency in performance of the magnetic sifter system and this protocol. Bootstrap-based approximately unbiased (AU) probability values were obtained for these two clusters, with the putative H1650 cluster having an AU of 100 (p < 0.05) and the putative H1975 cluster having an AU of 100 (p < 0.05) [48]. The two cell lines can thus be independently identified from the simulated heterogeneous mixture via either gene expression analysis or mutational analysis in a statistically significant manner.

## Discussion

In this work, we demonstrated high efficiency capture and release of rare cells with the high-throughput magnetic sifter and highly specific magnetic nanoparticle-antibody conjugates, and its ability to integrate well with a full-length mRNA-seq chemistry. While the overall yield is still non-ideal, the majority of losses are actually downstream of the magnetic sifter. The downstream FACS and Smart-seq2 chemistry-based sequencing are both commercial tools and were implemented here with standard protocols, and user optimization should further improve the yield from this method. Also, the modularity of this protocol facilitates the use of alternative single-cell cDNA synthesis chemistries or devices besides FACS and Smart-seq2 to improve the protocol yield further. These alternatives are an active area of research currently, and can include other microfluidic devices such as CytoSeq for high-throughput gene expression cytometry or droplet-based barcoding techniques for single-cell transcriptomics [49–51]. Other commercial solutions for improving the throughput of single-cell analysis include 10X Genomics’ GemCode platform, and Fluidigm C1-based 800 cell HT chip, however, these solutions still lack the capability to handle rare cells like CTCs in a complex background like blood, and indeed, would integrate well with the magnetic sifter in place of FACS and Smart-seq2 microplate-based chemistry.

It should be noted that a full-length mRNA-seq method (Smart-seq2) was chosen here for 2 reasons. Firstly, Ramskold et al. previously reported the successful application of this method in the sequencing of single CTC, and this chemistry is also commonly used on the Fluidigm C1 platforms for single-cell mRNA-seq, providing confidence in the robustness and quality of this chemistry [42]. Additionally, the use of full-length mRNA-seq methods provides certain advantages in analysis. Smart-seq2 has previously been shown to provide efficient detection of transcript variants and alleles due to its coverage, and reduced 3’-end bias [42, 52]. This is especially critical in human transcriptomic analysis, as most multi-exon genes in humans exhibit multiple isoforms and splice variants [53]. Recent work by Ziegenhain et al has also shown that Smart-Seq2 is most sensitive and provides the most even coverage of transcripts in a direct comparison with other single-cell RNA-seq methods like CEL-seq2, Drop-seq, MARS-seq and SCRB-seq, while remaining cost-effective for small cell numbers [54–57].

More critically, our mutational analysis of the H1650 full-length mRNA-seq data also suggests that in an actual biological sample, the use of the Smart-seq2 protocol in combination with the magnetic sifter can provide information for clonal analysis and lineage tracing, or for dissecting genetic heterogeneity in rare cells such as circulating tumor cells [58], even while conventional phenotype data is collected. This can also be useful in situations such as monitoring the development of resistance in cancer therapy, where single-cell heterogeneity is particularly relevant. As demonstrated in our simulated experiments, single cell heterogeneity can occur on both the genetic (H1650 vs H1975) and the phenotypic (CTC vs WBC) level, and this can be an approach to maximize information collection in both areas.

## Conclusions

Taken together, these experiments all demonstrate the ability of the magnetic sifter and magnetic nanoparticles to integrate with Smart-seq2 to provide high-quality transcriptomic data. Also, we derived a heuristic for analyzing the gene expression data for mutational information, and successfully demonstrated the ability to interrogate single-cell heterogeneity in a simulated CTC sample based on expression and mutational data.

## Methods

### Spiked CTC experiments

All cell lines were obtained from ATCC (Manassas, VA, USA). Both cell lines (NCI-H1650 and NCI-H1975) were maintained in RPMI-1640 media supplemented with 10% fetal bovine serum (FBS), 0.05 mg/mL penicillin, 0.05 mg/mL streptomycin, 2 mM GlutaMAX, 1 mM sodium pyruvate, and 0.1 mM MEM non-essential amino acids. All cell lines were maintained in an incubator at 37°C in 5% CO_2_.

For evaluation of tumor cell line capture efficiencies, the respective tumor cell lines are labeled with Green CellTracker CMFDA dye (Invitrogen, Carlsbad, CA, Catalog number: C7025), as per the product protocol, prior to detachment from the tissue culture plates. These fluorescently labeled cells are subsequently spiked into a 2 mL volume of healthy donor blood obtained from the Stanford Blood Center, followed by a 2-fold dilution in labeling buffer, and the addition of 100 μL of 0.5 mg/mL of anti-EpCAM functionalized magnetic nanoparticles (NVIGEN, Inc, Sunnyvale, CA). To obtain accurate counts of the number of spiked cells, a small droplet (≈ 1 μL) of tumor cell suspension is pipetted onto the inside of a micro-centrifuge tube cap, and all cells in the droplet are counted before the same cap is used to seal the micro-centrifuge tube containing the blood and the solution is mixed. Mixing is done under constant rotation for 1 hour at 4°C, whereupon the sample is processed through the magnetic sifter. After processing, the magnetic sifter is examined under a fluorescence microscope, and the capture efficiency is determined by counting the number of tumor cells on the surface and dividing this by the number of cells initially spiked.

A similar protocol is used for determining the harvest efficiency. However, subsequent to enumeration of the number of captured cells on the sifter, 400 μL of buffer is used to wash the cells off the magnetic sifter without any external applied magnetic field, and the eluted volume is spun onto a glass slide. The cells obtained are then counted by fluorescence microscope, and the harvest efficiency is obtained by dividing the number of cells counted on the glass slide by the number counted on the chip.

For the experiments which proceeded through to FACS and sequencing, to better simulate a real sample where fluorescence staining post-isolation is required, no CellTracker fluorescence staining was incorporated prior to spiking into blood. However, this made visual counting of the number of cells spiked into each experiment impractical. Hence, the concentration of the original cell suspension was counted via a hemocytometer 3 times, and an average was obtained. An appropriate volume as required to obtain the desired number of cells was then spiked directly into the donor blood sample without any visual counting.

### Cell immunostaining protocol

Cells were stained with a total of 4 reagents, 1 nuclear dye (Hoechst 33342), 2 antibodies against common blood cell markers (CD31 and CD45), and 1 antibody against an epithelial cell marker (EpCAM). Incubation was done simultaneously for all reagents, with the first 12 minutes at room temperature, and an additional 33 minutes on ice. Incubation was done with 5 μg/mL of Hoechst 33342 dye (Invitrogen, Carlsbad, CA, Catalog number: H3570) and 20x dilutions of APC-conjugated CD31 (Clone: WM59, Biolegend, Inc, San Diego, CA, Catalog number: 303116), APC-conjugated CD45 (Clone: HI30, Biolegend, Inc, San Diego, CA, Catalog number: 304012), and FITC-conjugated EpCAM (Clone: 9C4, Biolegend, Inc, San Diego, CA, Catalog number: 324204) antibodies. Upon completion of the incubation, cells are washed with buffer once, before being left on ice prior to FACS processing

### FACS protocol

All FACS sorts were done on a Sony LE-SH800 cell sorter (Sony Biotechnology Inc, San Jose, CA) with a 100 μm sorting chip (Catalog number: LE-C3110). Prior to starting the sort, the cell sorter and chip were calibrated with SH800 setup beads (Catalog number: LE-B3001) and fluorescence compensation was done with BD’s CompBeads (BD Biosciences, Franklin Lakes, NJ, Catalog number: 552843), incubated with the relevant fluorophore-conjugated antibodies (typically fluorescein isothiocyanate [FITC] and allophycocyanin [APC]).

Also, for each experiment, a 100 uL aliquot of blood from the original sample was lysed in an ammonium chloride-based red blood cell lysis buffer [59], stained with the same set of antibodies, and analyzed by FACS at the beginning to act as a negative control, and assist with the demarcation of gates to exclude blood cells for the actual samples of interest.

After the gates for the identification of blood cells are drawn, 96-well PCR plates (Bio-Rad Laboratories, Inc, Hercules, CA, Catalog number: HSP9601) are loaded onto the LE-SH800 cell sorter, and gated single cells are sorted into the wells at the purity set. Two purity settings were tested in this work, the “Ultra-Purity” and “Semi-Purity” mode, and it should be noted that the purity settings would affect cell yield, and the possibility of obtaining more than a single cell per droplet sorted.

The 96-well PCR plates were pre-loaded with a lysis buffer consisting of 0.1% Triton X-100 solution, 1 U/μL of RNAse inhibitor, and 2.5 μM of oligo-dT primer, and were spun down upon sort completion [30]. The PCR plates were also kept on dry ice between sorts, and while preparing for reverse transcription.

The FACS sort efficiencies were determined by dividing the number of positive events successfully sorted into the 96-well plate by the total number of positive events in the entire volume processed. Positive events in both instances are defined by their location within the gates drawn.

### mRNA-seq protocol

Single-cell full-length mRNA-seq was carried out as per the Smart-seq2 method detailed by Picelli et al [30]. The protocol was not adjusted, although some of the reagents were purchased from different vendors. A complete list of the reagents and vendors are detailed in Table 1, although the complete protocol is not reproduced here for brevity. After reverse transcription and amplification, cDNA generated from each single-cell was checked for quality on a fragment analyzer (Advanced Analytical Technologies, Inc, Ankeny, IA). Selected single-cells then underwent library preparation for Illumina sequencing with the Nextera XT DNA library preparation kit (Illumina, Inc, San Diego, CA). Multiplexed library pools were then pooled and sequenced as 75bp paired-end Illumina reads utilizing the NextSeq 500 High Output Kit v2.

**Table 1:** List of reagents used for Smart-seq2 and their respective vendors and catalog numbers.

Reagent	Vendor	Catalog Number
Oligo-dT Primer	IDT Technologies	Custom-order
ISPCR Primer	IDT Technologies	Custom-order
TSO Oligonucleotide	Exiqon	Custom-order
Recombinant RNAse Inhibitor	Clontech Laboratories	2313A
Episcript RNAse H- Reverse Transcriptase	Epicentre	ERT 12925K
Betaine (5M)	Affymetrix	77507
dNTP Mix (10 mM)	Thermo Fisher Scientific	R0192
Magnesium Chloride	Thermo Fisher Scientific	AM9430G
KAPA HiFi HotStart ReadyMix (2X)	KAPA Biosystems	KK2602
Agencourt Ampure XP Beads	Beckman Coulter	A63881
Nextera XT DNA Library Preparation Kit	Illumina	FC-131-1096

The Smart-seq2 cDNA synthesis efficiency was determined by dividing the number of wells in the PCR micro-plates processed that produced any cDNA profile (including both good-quality cDNA profiles and degraded cDNA profiles) by the putative number of cells successfully sorted by FACS. It should be noted that this measure could be an underestimate if the gates contain other cellular debris that have the same scattering and fluorescence profiles as the cell lines. However, negative control experiments comprising 50 μL of healthy donor blood without any spiked cells showed no positive signals from gates that were similarly drawn.

### Data analysis

The reads were preprocessed using Prinseq [**prinseq**.sourceforge.net/] to filter away short reads shorter than 30, followed by trimming of the first 10 bp on the 5’-end and trimming of reads with low quality on the 3’-end. Low complexity reads are then removed using (-lc_method entropy \-lc_threshold 65).

We then used FASTQC [http://www.bioinformatics.babraham.ac.uk/projects/fastqc] to determine overrepresented sequences and removed those using cutadapt [https://cutadapt.readthedocs.org/en/stable/]. Next, we used Prinseq to remove orphan pairs less than 30bp in length before removal of Nextera adapters via Trim Galore [http://www.bioinformatics.babraham.ac.uk/projects/trim_galore/].

Remaining reads were aligned to the hg19 genome with TOPHAT. After alignment of the reads, read sequences were analyzed for gene expression levels by Cufflinks (expression level data in FPKM) and HT-seq (expression level data in counts/gene) [60, 61].

Mutational analysis was done via the bam-readcount package from https://github.com/genome/bam-readcount. A list of SNPs was obtained from COSMIC and base counts for relevant genome locations were obtained via the bam-readcount package.

The mRNA-Seq data from this study has been deposited in the NCBI sequence read archive under the study accession number SRP107036, with the bam files spanning accession numbers SRR5556747-SRR5556840.

## Supporting information

S1 Table List of SNPs observed in the H1650 cells sequenced. Genes are listed in the same order as per the axis in Fig 4 when read from left to right.
(DOCX)

S2 Table List of SNPs observed in the H1650 cells sequenced. Genes are listed in the same order as per the axis in Fig 5 when read from left to right.
(DOCX)

S3 Table List of SNPs observed in the H1650 and H1975 cells sequenced. Genes are listed in the same order as per the axis in Fig 7 when read from left to right.
(DOCX)
