# Supplementary material for: High-throughput full-length single-cell mRNA-seq of rare cells
Source: PLoS One. 2017 Nov 29;12(11):e0188510. doi: 10.1371/journal.pone.0188510 (PMC5706670; doi:10.1371/journal.pone.0188510)
Supplement: S2 Table — Genes are listed in the same order as per the axis in Fig 5 when read from left to right. (DOCX) [file pone.0188510.s002.docx]

| **Gene** | **Mutation** | **Chr** | **Base Pair Location** |
| --- | --- | --- | --- |
| BRIX1 | BRIX1:c.32G>A | chr5 | 34915875 |
| SBDS | SBDS:c.397A>G | chr7 | 66458266 |
| CASP4 | CASP4:c.644G>A | chr11 | 104820407 |
| MMD | MMD:c.144C>T | chr17 | 53488743 |
| HLA-C | HLA-C_ENST00000539307:c.466C>T | chr6 | 31239114 |
| IFRD1 | IFRD1:c.939G>A | chr7 | 112108068 |
| DEK | DEK:c.663A>G | chr6 | 18249981 |
| DBNL | DBNL:c.271G>A | chr7 | 44092484 |
| C8orf47 | C8orf47:c.814G>C | chr8 | 99102059 |
| C8orf47 | C8orf47:c.151G>C | chr8 | 99101396 |
| ANLN | ANLN:c.3320T>C | chr7 | 36492154 |
| DSN1 | DSN1:c.484G>A | chr20 | 35395190 |
| MTHFS | MTHFS:c.355G>A | chr15 | 80181459 |
| OXSM | OXSM:c.1035C>T | chr3 | 25835640 |
| GCAT | GCAT:c.669A>G | chr22 | 38211225 |
| SFPQ | SFPQ:c.1953A>T | chr1 | 35652635 |
| EIF4G1 | EIF4G1:c.3819C>T | chr3 | 184045656 |
| OMG | OMG:c.32G>A | chr17 | 29623318 |
| TCTN3 | TCTN3_ENST00000265993:c.998C>A | chr10 | 97444353 |
| GLUD1 | GLUD1:c.832C>T | chr10 | 88822502 |
| FAM86A | FAM86A:c.791G>A | chr16 | 5139209 |
| FKBP9 | FKBP9:c.231A>G | chr7 | 33014238 |
| TOR1AIP2 | TOR1AIP2:c.540A>G | chr1 | 179819993 |
| SRRM1 | SRRM1_ENST00000447431:c.1637A>G | chr1 | 24989688 |
| DFNA5 | DFNA5:c.68C>T | chr7 | 24789326 |
| INTS10 | INTS10:c.600C>A | chr8 | 19680888 |
| NIPBL | NIPBL:c.7911G>A | chr5 | 37063942 |
| DIDO1 | DIDO1:c.2314G>A | chr20 | 61526418 |
| CRLS1 | CRLS1:c.439G>A | chr20 | 5990553 |
| CRLS1 | CRLS1:c.575-3C>G | chr20 | 6011928 |
| EFHD1 | EFHD1:c.226A>T | chr2 | 233498640 |
| ECHDC3 | ECHDC3:c.680C>T | chr10 | 11805311 |
| RPIA | RPIA:c.680G>A | chr2 | 89036135 |
| NPIPL3 | ENSG00000185864_ENST00000357370:c.953C>T | chr16 | 21848641 |
| CWF19L2 | CWF19L2:c.1009G>A | chr11 | 107288976 |
| MFN2 | MFN2_ENST00000376337:c.1256T>G | chr1 | 12073412 |
| ZNF562 | ZNF562_ENST00000453372:c.339G>A | chr19 | 9767232 |
| ZFP64 | ZFP64_ENST00000361387:c.356A>T | chr20 | 50782495 |
| LAMC1 | LAMC1:c.2853C>T | chr1 | 183095306 |
| CDK11A | CDC2L2:c.319G>T | chr1 | 1650803 |
| KIAA0907 | KIAA0907:c.991A>C | chr1 | 155891673 |
| RAPH1 | RAPH1_ENST00000308091:c.937G>C | chr2 | 204326600 |
| ASH1L | ASH1L:c.2232C>T | chr1 | 155450429 |
| PCNT | PCNT:c.9280G>C | chr21 | 47860002 |
| SMARCA2 | SMARCA2:c.2992-4T>G | chr9 | 2097381 |
| ST5 | ST5:c.3063C>T | chr11 | 8718203 |
| TACC1 | TACC1:c.230C>T | chr8 | 38646290 |
| MYO9B | MYO9B:c.2107C>T | chr19 | 17283739 |
| ADAMTS12 | ADAMTS12_ENST00000515401:c.289C>T | chr5 | 33881424 |
| ZNF724P | ZNF724P:c.1154A>G | chr19 | 23405893 |
| COBRA1 | COBRA1:c.363C>T | chr9 | 140150877 |
| HIST1H2BF | HIST1H2BF:c.102C>G | chr6 | 26199888 |
| ZNF543 | ZNF543:c.1709G>A | chr19 | 57840539 |
| ZNF543 | ZNF543:c.1765G>C | chr19 | 57840595 |
| PIK3C2A | PIK3C2A:c.4570T>C | chr11 | 17113615 |
| C1R | C1R:c.920G>A | chr12 | 7241473 |
| FAM69B | FAM69B:c.369C>T | chr9 | 139616639 |
| HGSNAT | HGSNAT:c.708G>A | chr8 | 43052138 |
| MFSD2A | MFSD2A:c.207C>T | chr1 | 40422872 |
| POLQ | POLQ:c.6805G>A | chr3 | 121195392 |
| ELMO3 | ELMO3:c.2092G>A | chr16 | 67237469 |
| DENND4A | DENND4A_ENST00000443035:c.925A>G | chr15 | 66030160 |
| CYP2R1 | CYP2R1:c.286A>C | chr11 | 14907403 |
| ZC3H7B | ZC3H7B:c.671C>T | chr22 | 41735050 |
| TMEM63B | TMEM63B:c.1386C>T | chr6 | 44116655 |
| ZNF254 | ZNF254_ENST00000357002:c.423A>T | chr19 | 24309225 |
| RLTPR | RLTPR_ENST00000334583:c.4165A>G | chr16 | 67691189 |
| PGM5 | PGM5:c.869C>T | chr9 | 71006621 |
| MLL2 | MLL2_ENST00000301067:c.2663_2665delAAC | chr12 | 49444801 |
| KCTD21 | KCTD21:c.369C>T | chr11 | 77885232 |
| FBXW10 | FBXW10:c.2325C>A | chr17 | 18678495 |
| ANKRD26P1 | FLJ43980:c.813A>G | chr16 | 46562244 |
| KHDRBS3 | KHDRBS3:c.958G>C | chr8 | 136659244 |
| ALDH1L1 | ALDH1L1:c.1036G>A | chr3 | 125865718 |
| KIAA1614 | KIAA1614:c.1179C>T | chr1 | 180897683 |
| KRT86 | KRT86:c.800G>A | chr12 | 52699088 |
| ERG | ERG_ENST00000417133:c.287A>G | chr21 | 39795454 |
| ADAMTSL2 | ADAMTSL2:c.2325C>G | chr9 | 136434610 |
| CACNA1B | CACNA1B:c.639C>T | chr9 | 140809122 |
| ABCG8 | ABCG8:c.1453G>A | chr2 | 44101587 |

**S2 Table. List of SNPs observed in the H1650 cells sequenced. Genes are listed in the same order as per the axis in Fig 5. when read from left to right.**
