# Supplementary material for: High-throughput full-length single-cell mRNA-seq of rare cells
Source: PLoS One. 2017 Nov 29;12(11):e0188510. doi: 10.1371/journal.pone.0188510 (PMC5706670; doi:10.1371/journal.pone.0188510)
Supplement: S3 Table — Genes are listed in the same order as per the axis in Fig 7 when read from left to right. (DOCX) [file pone.0188510.s003.docx]

| **Gene** | **Mutation** | **Chr** | **Base Pair Location** |
| --- | --- | --- | --- |
| SKA3 | SKA3_ENST00000400018: c.276A>G | chr13 | 21746533 |
| CCDC50 | CCDC50: c.318G>T | chr3 | 191078936 |
| GUK1 | GUK1_ENST00000366722: c.474G>T | chr1 | 228336076 |
| NTPCR | NTPCR: c.12C>G | chr1 | 233086468 |
| IGF2BP3 | IGF2BP3: c.1009G>T | chr7 | 23383405 |
| GSTM3 | GSTM3: c.15G>A | chr1 | 110282895 |
| PSMD7 | PSMD7: c.348T>C | chr16 | 74335541 |
| EPCAM | EPCAM: c.501G>A | chr2 | 47604162 |
| POLR2E | POLR2E: c.288G>A | chr19 | 1091851 |
| EIF3G | EIF3G: c.219T>C | chr19 | 10229565 |
| DCPS | DCPS: c.432G>T | chr11 | 126201355 |
| SERINC3 | SERINC3: c.1259T>C | chr20 | 43129738 |
| POLR2B | POLR2B: c.65G>T | chr4 | 57852565 |
| LONP2 | LONP2: c.2299A>C | chr16 | 48382163 |
| GLMN | GLMN: c.510C>A | chr1 | 92754593 |
| PIGV | PIGV: c.1222T>A | chr1 | 27124075 |
| EGFR L858R | EGFR: c.2573T>G | chr7 | 55259515 |
| EGFR T790M | EGFR: c.2369C>T | chr7 | 55249071 |
| CD82 | CD82_ENST00000227155: c.245G>A | chr11 | 44626716 |
| XAB2 | XAB2: c.1588G>A | chr19 | 7687246 |
| PIGX | PIGX: c.498G>A | chr3 | 196457951 |
| FHL2 | FHL2: c.296C>G | chr2 | 105990051 |
| CTNNA1 | CTNNA1: c.2238C>G | chr5 | 138266564 |
| TNNT1 | TNNT1: c.779A>G | chr19 | 55645267 |
| PDXDC1 | PDXDC1: c.1391C>T | chr16 | 15123905 |
| STK4 | STK4: c.301G>A | chr20 | 43610525 |
| UBR1 | UBR1: c.410G>A | chr15 | 43374843 |
| LEO1 | LEO1: c.1906C>T | chr15 | 52230448 |
| NEDD1 | NEDD1_ENST00000557644: c.1007A>T | chr12 | 97331040 |
| CTTN | CTTN_ENST00000376561: c.614A>C | chr11 | 70265897 |
| SIKE1 | SIKE1_ENST00000369528: c.455T>C | chr1 | 115319046 |
| RAP1GDS1 | RAP1GDS1: c.1529C>T | chr4 | 99355175 |
| C1orf112 | C1orf112: c.188T>A | chr1 | 169772326 |
| OBFC1 | OBFC1: c.469G>A | chr10 | 105658747 |
| FKBP9 | FKBP9:c.231A>G | chr7 | 33014238 |
| ELMO3 | ELMO3:c.2092G>A | chr16 | 67237469 |
| ZNF737 | ZNF737: c.621A>G | chr19 | 20728385 |
| HLA-C | HLA-C_ENST00000539307:c.466C>T | chr6 | 31239114 |
| COX10 | COX10: c.34C>G | chr17 | 13972956 |
| EDEM2 | EDEM2: c.1259_1260insCAA | chr20 | 33703713 |
| APOBEC3B | APOBEC3B: c.966G>T | chr22 | 39387579 |
| PSMD1 | PSMD1: c.1823_1824delGT | chr2 | 231951835 |
| ENSG00000121031 | ENSG00000121031: c.11505G>A | chr8 | 48691636 |
| CRLS1 | CRLS1:c.439G>A | chr20 | 5990553 |
| GBA | GBA: c.772G>T | chr1 | 155207359 |
| ECOP | ECOP: c.260C>T | chr7 | 55540694 |
| ENSG00000172261 | ENSG00000172261: c.513G>C | chr20 | 47751529 |
| RPIA | RPIA:c.680G>A | chr2 | 89036135 |
| C8orf47 | C8orf47:c.151G>C | chr8 | 99101396 |
| C8orf47 | C8orf47:c.814G>C | chr8 | 99102059 |
| HLA-C | HLA-C: c.560C>A | chr6 | 31238909 |
| CASP4 | CASP4:c.644G>A | chr11 | 104820407 |
| MRPS5 | MRPS5: c.541C>T | chr2 | 95774016 |
| MTHFS | MTHFS:c.355G>A | chr15 | 80181459 |
| TOR1AIP2 | TOR1AIP2:c.540A>G | chr1 | 179819993 |
| DBNL | DBNL:c.271G>A | chr7 | 44092484 |
| SBDS | SBDS:c.397A>G | chr7 | 66458266 |
| ANLN | ANLN:c.3320T>C | chr7 | 36492154 |
| DSN1 | DSN1:c.484G>A | chr20 | 35395190 |
| OXSM | OXSM:c.1035C>T | chr3 | 25835640 |
| BRIX1 | BRIX1:c.32G>A | chr5 | 34915875 |
| IFRD1 | IFRD1:c.939G>A | chr7 | 112108068 |
| ZNF562 | ZNF562_ENST00000453372:c.339G>A | chr19 | 9767232 |
| MMD | MMD:c.144C>T | chr17 | 53488743 |
| TCTN3 | TCTN3_ENST00000265993:c.998C>A | chr10 | 97444353 |
| SFPQ | SFPQ:c.1953A>T | chr1 | 35652635 |
| CRLS1 | CRLS1:c.575-3C>G | chr20 | 6011928 |
| FAM86A | FAM86A:c.791G>A | chr16 | 5139209 |
| ZNF737 | ZNF737: c.840A>G | chr19 | 20728166 |
| CDC2L2 | CDC2L2:c.319G>T | chr1 | 1650803 |
| DIDO1 | DIDO1:c.2314G>A | chr20 | 61526418 |
| MFN2 | MFN2_ENST00000376337:c.1256T>G | chr1 | 12073412 |
| GLUD1 | GLUD1:c.832C>T | chr10 | 88822502 |
| INTS10 | INTS10:c.600C>A | chr8 | 19680888 |
| EIF4G1 | EIF4G1:c.3819C>T | chr3 | 184045656 |
| DEK | DEK:c.663A>G | chr6 | 18249981 |

**S3 Table. List of SNPs observed in the H1650 and H1975 cells sequenced. Genes are listed in the same order as per the axis in Fig 7. when read from left to right.**
